# Identification of Heterozygous Single- and Multi-exon Deletions in *IL7R* by Whole Exome Sequencing

**DOI:** 10.1007/s10875-016-0343-9

**Published:** 2016-11-02

**Authors:** Karin R. Engelhardt, Yaobo Xu, Angela Grainger, Mila G. C. Germani Batacchi, David J. Swan, Joseph D. P. Willet, Intan J. Abd Hamid, Philipp Agyeman, Dawn Barge, Shahnaz Bibi, Lucy Jenkins, Terence J. Flood, Mario Abinun, Mary A. Slatter, Andrew R. Gennery, Andrew J. Cant, Mauro Santibanez Koref, Kimberly Gilmour, Sophie Hambleton

**Affiliations:** 1 Primary Immunodeficiency Group, Institute of Cellular Medicine, Newcastle University, Newcastle upon Tyne, UK; 2 Institute of Genetic Medicine, Newcastle University, Newcastle upon Tyne, UK; 3 Great North Children’s Hospital, Newcastle upon Tyne Hospitals NHS Foundation Trust, Newcastle upon Tyne, UK; 4 NE Thames Regional Genetics Service, Great Ormond Street Hospital NHS Foundation Trust, London, UK; 5 Immunology, Great Ormond Street Hospital NHS Foundation Trust, London, UK

**Keywords:** IL7R, copy number variation, compound heterozygous, SCID, whole exome sequencing

## Abstract

**Purpose:**

We aimed to achieve a retrospective molecular diagnosis by applying state-of-the-art genomic sequencing methods to past patients with T-B+NK+ severe combined immunodeficiency (SCID). We included identification of copy number variations (CNVs) by whole exome sequencing (WES) using the CNV calling method ExomeDepth to detect gene alterations for which routine Sanger sequencing analysis is not suitable, such as large heterozygous deletions.

**Methods:**

Of a total of 12 undiagnosed patients with T-B+NK+ SCID, we analyzed eight probands by WES, using GATK to detect single nucleotide variants (SNVs) and small insertions and deletions (INDELs) and ExomeDepth to detect CNVs.

**Results:**

We found heterozygous single- or multi-exon deletions in *IL7R*, a known disease gene for autosomal recessive T-B+NK+ SCID, in four families (seven patients). In three families (five patients), these deletions coexisted with a heterozygous splice site or nonsense mutation elsewhere in the same gene, consistent with compound heterozygosity. In our cohort, about a quarter of T-B+NK+ SCID patients (26%) had such compound heterozygous *IL7R* deletions.

**Conclusions:**

We show that heterozygous *IL7R* exon deletions are common in T-B+NK+ SCID and are detectable by WES. They should be considered if Sanger sequencing fails to detect homozygous or compound heterozygous *IL7R* SNVs or INDELs.

**Electronic supplementary material:**

The online version of this article (doi:10.1007/s10875-016-0343-9) contains supplementary material, which is available to authorized users.

## Introduction

Whole exome sequencing (WES) is a powerful tool for discovering pathogenic variants in rare genetic diseases, detecting both single nucleotide variants (SNVs) and small insertions/deletions in known disease genes with relative efficiency. WES has not been seen as offering equivalent advantages for the detection of copy number variations (CNVs), which instead may be sought by chromosomal microarrays or multiplex ligation-dependent probe amplification (MLPA). However, recent computational advances have also made it possible to identify CNVs by WES [1, 2]. This is of great advantage as limitations of array-based CNV detection methods, such as noisy signal and low resolution, make detection of small CNVs difficult, and large heterozygous deletions can only be detected by conventional PCR-based sequencing methods using genomic DNA if the breakpoints of the deletion are known. Therefore, routine Sanger sequencing analysis is not suitable for the detection of such gene alterations.

In our center, we have treated a cohort of 19 patients with T-B+NK+ severe combined immunodeficiency (SCID), characterized by early symptoms of immunodeficiency (such as failure to thrive and recurrent infections), and/or a positive family history of T-B+NK+ SCID, together with profound T lymphocytopenia, low serum immunoglobulin levels and impaired lymphocyte proliferation to mitogens. Autosomal recessive (AR) mutations in several genes have been reported to cause T-B+NK+ SCID. Amongst these are *CD3E*, *CD3D* and *CD3Z*, all of which encode subunits of the CD3/T cell receptor complex [3, 4], as well as *IL7R* [5], which encodes IL7Rα, commonly known as IL7R, the unique alpha chain of the heterodimeric receptor for interleukin-7 (IL-7). IL-7 is necessary for T lymphocyte development in the thymus and for proliferation and survival of T lymphocytes in the periphery [6, 7]. Coronin-1A deficiency due to AR mutations in *CORO1A* can cause T-B+NK+ SCID through impaired actin cytoskeleton regulation [8–10]. Coronin-1A is an actin-binding protein required for lymphocyte migration and thymic egress. The human Nude/T-B+NK+ SCID phenotype is caused by mutations in the gene *FOXN1*, which encodes a transcription factor crucial for thymus development [11, 12]. An autosomal dominant T cell differentiation defect due to congenital absence of the thymus can be found in ‘complete’ chromosome 22q11.2 deletion syndrome (DiGeorge syndrome), in which the immunodeficiency is usually part of a clinical triad also including congenital cardiac and parathyroid gland defects [13].

**Fig. 1:**
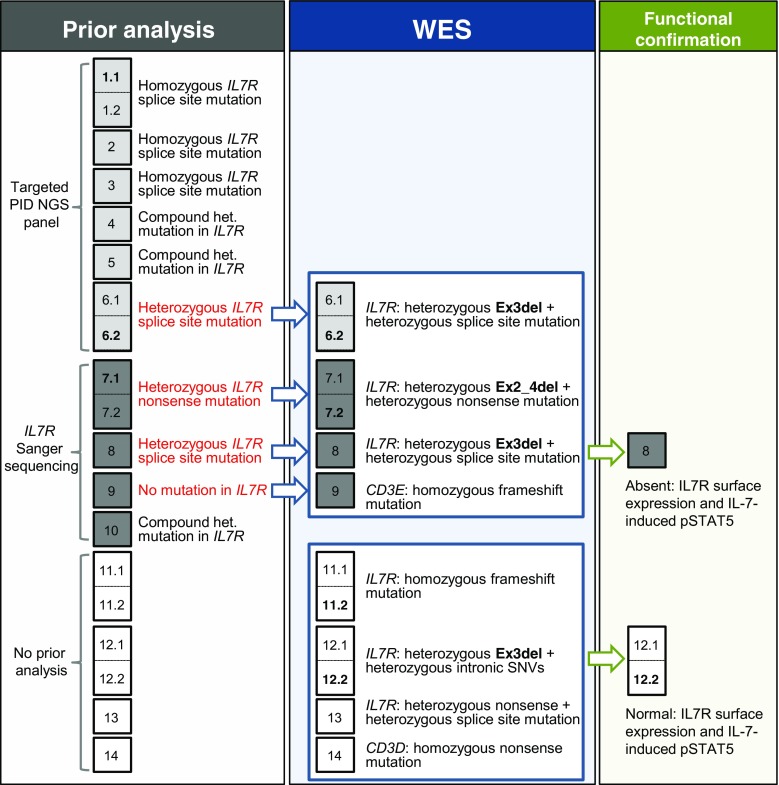
Workflow and findings of patient analysis. The *first column* shows molecular analysis before whole exome sequencing (WES) for 19 patients from 14 families. *Rectangles* contain either single patients (x) or siblings (x.1, x.2). If in a family only one sibling was analyzed, his or her number is shown in *bold*. Patients who had either not been previously analyzed or for whom no causative mutation had been found (findings shown in *red*) were subjected to WES (*second column*). For two patients, cryopreserved PBMCs were available for functional testing (*third column*). *Het.* heterozygous; *Ex3del* deletion of exon 3; *Ex2_4del* deletion of exons 2-4

IL7R deficiency had been diagnosed in seven patients of our cohort of non-syndromic T-B+NK+ SCID by conventional methods (Fig. 1). The remaining 12 patients from eight families were screened by WES to identify disease-causing mutations, resulting in the molecular diagnosis of ten patients.

Here, we show successful detection by WES of heterozygous single- or multi-exon deletions in the gene *IL7R*, invisible by conventional sequencing techniques, in patients with T-B+NK+ SCID.

## Methods

### Study Subjects

Patients and their relatives provided written informed consent to participate in research protocols approved by the Newcastle and North Tyneside 1 Research Ethics Committee. Whole blood samples, buccal samples or dermal fibroblast cultures were obtained from these individuals, and genomic DNA was isolated using the DNeasy or QIAamp DNA mini kit (Qiagen).

### PCR and Sequencing Analysis

Specific primers were designed in Primer3web version 4.0.0 (http://bioinfo.ut.ee/primer3/). Primer sequences are available on request. Capillary sequencing was performed according to standard methods. Sequences were aligned with the consensus coding sequence (human genome assembly 37 in nucleotide BLAST (http://blast.ncbi.nlm.nih.gov/blast/). ChromasLite Version 2.1.1 was used for visualization of the sequences.

### WES and CNV Analysis

Whole exomes of the patients were enriched with the Agilent SureSelect Human All Exon V5 kit (Santa Clara, CA, USA) and subsequently sequenced using the Illumina HiSeq 2500 sequencing system (San Diego, CA, USA) by AROS (Applied Biotechnology AS, Denmark). Sequencing reads were analyzed using the following workflow to identify variants in patients. Firstly, the quality of sequencing reads was checked with FastQC (http://www.bioinformatics.babraham.ac.uk/projects/fastqc/). Duplicated reads were removed with FastUniq [14]. The remaining reads were mapped to the human reference genome GRCh37 with BWA [15]. The alignments were refined with tools of the GATK suite [16]. Variants were called according to GATK Best Practice recommendations [17, 18], including recalibration, as well as using Freebayes [19]. The variants called by Freebayes with total coverage ≥5, minor allele coverage ≥5 and variant call quality ≥20 were added to those identified by GATK. Non-synonymous exonic variants were subsequently filtered by quality and minor-allele frequency (MAF) reported in the 1000 Genomes project (2012 Feb release) [20] and ESP6500 [21]. Variants with MAF >0.05 were excluded. Annovar was used for annotations and prediction of functional consequences [22]. Copy number variants (CNVs) were called using ExomeDepth from the GATK refined alignments [1].

### Lymphocyte Proliferation

Lymphocyte proliferation in vitro to mitogen phytohaemagglutinin (PHA) was assayed by tritiated thymidine incorporation in an accredited clinical laboratory using standard techniques.

#### IL-7Rα Expression

Five microliters of anti-human CD127 PE (IL7Rα) (BD Biosciences) or matched isotype control (BD Biosciences) was added to 100 μl of blood or 10^6^ PBMCs, incubated for 10 min at room temperature. Cells were lysed with FACsLyse (BD Biosciences) for 10 min, and the remaining cells were washed with Cell Wash (BD Biosciences) before being fixed (FACsFix, BD Bioscience). Ten thousand lymphocytes were acquired (FACsCalibur, BD Biosciences) and analyzed (Cell Quest Pro, BD Biosciences).

#### STAT5 Phosphorylation Assay

1 × 10^4^ IU/ml IL-2 (Chiron), 50 ng/ml IL-15 (R&D systems) or 10 ng/ml IL-7 (R&D Systems) were added to 100 ml of whole blood or 10^6^ PBMCs and placed at 37°C for 10 min to stimulate cells. Two milliliters of prewarmed FACs Lyse/Fix (BD Biosciences) were then added to the cells, mixed and placed at 37°C for 10 min. The cells were pelleted and washed once with STAT wash (phosphate-buffered saline containing 1 % fetal calf serum). The cells were resupended in cold Perm Buffer III (BD Biosciences) and placed at 4°C for 30 min. The cells were then washed once with STAT wash before 5 ml of antibodies (STAT5 ptyr, and CD4 PerCP (BD Biosciences) were added, and the cells were incubated at room temperature for 30 min in the dark, washed with STAT wash and fixed (FACsFix, BD Biosciences). Ten thousand lymphocyte events were acquired (FACsCalibur, BD Biosciences) and analyzed using CellQuest software (BD Biosciences).

## Results

### Case Summaries

Patient 6.1 presented at age 3 months with diarrhea and failure to thrive (Table 1). Diagnosis was made at the age of 10 months, when he additionally presented with superficial candidiasis and lymphocytopenia with absent T lymphocytes. Patient 6.2 is the sister of patient 6.1 and was diagnosed shortly after birth because of family history, low lymphocyte numbers and weak proliferative responses to mitogen. Patient 7.1 presented with recurrent febrile episodes from the age of 1 month. At 7 months, persistent rotavirus gastroenteritis and weight loss were documented. At 8 months, he was admitted to hospital with pneumocystis jirovecii pneumonia and the diagnosis of SCID was made. Patient 7.2 is the brother of patient 7.1 and was diagnosed shortly after birth because of family history, absent T cells and low lymphocyte proliferation. Patient 8 presented at the age of 5 months with progressive cough, vomiting, poor appetite and then failure to thrive. Diagnosis was made at age of 8 months, when she was admitted to the hospital with pneumocystis jirovecii pneumonia and respiratory syncytial virus pneumonitis. Patient 12.1 presented at the age of 3 months with recurrent chest infections, oral candidiasis and failure to thrive. He was diagnosed as having SCID at the age of 8 months. Patient 12.2 is the brother of patient 12.1 and was diagnosed shortly after birth because of family history, absent T cells and low lymphocyte proliferation.

**Table 1:** Patients’ clinical and laboratory characteristics

	Family 6		Family 7		Family 8	Family 12	
Patient	6.1	6.2	7.1	7.2	8	12.1	12.2
Gender	M	F	M	M	F	M	M
Consanguinity	No	No	No	No	No	No	No
Age of first presentation (months)	3	NA	NA	6–7	8	3	NA
Age at diagnosis (months)	10	0	0	8	8	8	0
Diagnostic trigger	Failure to thrive	Family history	Family history	Recurrent infections	Failure to thrive, respiratory infection, lymphopenia	Recurrent infections, failure to thrive	Family history
Infections pre diagnosis	Candida, viral gastroenteritis	None	None	RSV bronchio-litis, persistent rotavirus, candida, PJP	RSV, PJP	Recurrent URTI, para-influenza type I, persistent candida	None
Other problems	Persistent diarrhea	None	None	Failure to thrive, encephalopathy	None	Possible encephalitis	None
Age at BMT (months)	12	1	2	NA	11	8 and 12	2
Status	Alive	Alive	Alive	Died pre transplant	Alive	Died 115 days post second transplant	Alive
Serum immunoglobulins at diagnosis (g/l) (normal ranges according to the Harriet Lane Handbook 19th edition)							
IgG	8.1 (2.9–10.7)	10.4 (6.4–16.1)	8.7 (6.4–16.1)	3 (2.2–9.0)	<0.4 (2.2–9.0)	<0.33 (2.2–9.0)	10.5 (6.4–16.1)
IgA	0.23 (0.16–0.84)	<0.01 (0.01–0.04)	<0.3 (0.01–0.04)	0.12 (0.11–0.90)	<0.3 (0.11–0.90)	<0.07 (0.11–0.90)	<0.07 (0.01–0.04)
IgM	1.71 (0.41–1.49)	0.18 (0.06–0.25)	<0.22 (0.06–0.25)	1.15 (0.34–1.26)	<0.19 (0.34–1.26)	<0.2 (0.34–1.26)	0.17 (0.06–0.25)
Lymphocyte subpopulations (cells/mm3) (normal ranges according to Comans-Bitter, J Pediatr 1997)							
CD3	185 (1600–6700)	0 (600–5000)	0 (600–5000)	0 (2400–6900)	0 (2400–6900)	10 (2400–6900)	0 (600–5000)
CD4	42 (1000–4600)	0 (400–3500)	0 (400–3500)	0 (1400–5100)	0 (1400–5100)	0 (1400–5100)	0 (400–3500)
CD8	144 (400–2100)	0 (200–1900)	0 (200–1900)	0 (600–2200)	0 (600–2200)	0 (600–2200)	0 (200–1900)
CD19	227 (600–2700)	24 (40–1100)	39 (40–1100)	1395 (700–2500)	362 (700–2500)	250 (700–2500)	627 (40–1100)
NK	52 (200–1200)	23 (100–1900)	832 (100–1900)	71 (100–1000)	161 (100–1000)	ND	697 (100–1900)
Lymphocyte proliferation (cpm) [SI]							
Patient PHA (control)	No response (ND)	217 [−] (49370 [117x])	ND	2336 [1x] (95381 [521x])	ND	138 [0.9x] (32003 [89x])	511 [0.3x] (95069 [211x])
ConA	97 (1824)	ND	ND	ND	ND	ND	ND
PWM	131 (4337)	ND	ND	ND	ND	ND	ND
Genetic analysis							
Prior to WES	No	Targeted PID panel	*IL7R* Sanger sequencing	No	*IL7R* Sanger sequencing	No	No
Results	NA	Het. *IL7R* c.221+2T>G	Het. IL7R p.Q26X	NA	Het. *IL7R* c.221+2T>G	NA	NA
WES	No	Yes	No	Yes	Yes	No	Yes
Results	NA	Het. Ex3del + het. c.221+2T>G	NA	Het. Ex2_4del + het. p.Q26X	Het. Ex3del + het. c.221+2T>G	NA	Het. Ex3del + intronic SNVs

*ConA* concanavalin A, *Het*. heterozygous, *NA* not applicable, *ND* not determined, *PHA* phytohemagglutinin, *PJP* pneumocystis jiroveci pneumonia, *PWM* poke weed mitogen, *RSV* respiratory syncytial virus, *SI* stimulation index (cpm of stimulated/cpm of unstimulated cells), *URTI* upper respiratory tract infection

### Whole Exome Sequencing Results

In two patients, we found homozygous loss-of-function mutations in CD3 chains. In patient 9, the mutation was in *CD3E* (c.424delG; p.G142fsX162), and in patient 14 in *CD3D* (c.202C>T; p.R68X). Two siblings had a homozygous frameshift deletion in *IL7R* (c.493delC; p.H165fsX167; family 11) (data not shown). These homozygous variants are predicted to be disease-causing in each case. Patient 13 had a compound heterozygous mutation in *IL7R* (heterozygous c.221+2T>G and heterozygous c.76C>T, p.Q26X) (data not shown). Several splice site prediction programs predict disruption of the exon 2 splice donor site due to the c.221+2T>G mutation (Table S1). Furthermore, a similar mutation (heterozygous c.221+2T>A) together with a heterozygous missense mutation in *IL7R* was found by Lee et al. in a patient with T-B+ SCID [23]. Thus, compound heterozygosity for these variants could be considered causative.

**Fig. 2:**
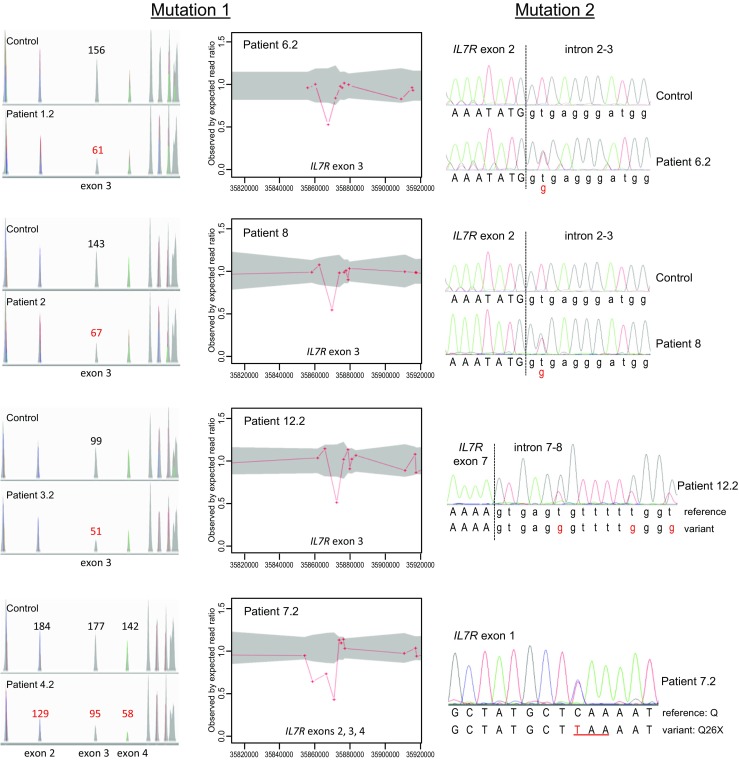
Compound heterozygous *IL7R* mutations. Mutation 1—hemizygosity of exon 3 or exons 2-4 demonstrated by ExomeDepth. Shown for each exon are peaks representing read depth (*left*) and the observed to expected ratio of reads (*right*). *Gray area* expected value range. Affected exons have fewer reads than those of other samples of the same batch. Mutation 2—Sanger sequencing of heterozygous SNVs (indicated in *red*)

WES sequencing of patients 6.2, 7.2, 8 and 12.2 from the remaining four kindreds revealed a previously undetected heterozygous deletion of one or three exons of *IL7R*, coexisting with heterozygous SNVs elsewhere in the same gene (Fig. 2). Patients 6.2 and 8 had a heterozygous deletion of exon 3 (Ex3del) together with a heterozygous exon 2 splice donor site mutation (c.221+2T>G). Patient 7.2 had a heterozygous deletion of exons 2-4 (Ex2_4del) together with a nonsense mutation in exon 1 (c.76C>A; p.Q26X). Patient 12.2 had a heterozygous exon 3 deletion (Ex3del) together with heterozygous SNVs at positions +6, +12 and +15 in the exon 7 splice donor site (c.876+6T>G; c.876+12T>G; c.876+15T>G). The breakpoints in patients 6.2 and 7.2 were determined by Sanger sequencing (Figure S1), confirming the WES finding of exon 3 or exon 2-4 hemizygosity, respectively. Unfortunately, we were unable to define the exon 3 deletion breakpoints in patients 8 and 12.2, which suggests that they are different from that of family 6. Each of these exonic deletions implies a frameshift with a premature stop codon: if mRNA were to escape nonsense-mediated decay, any protein product would be severely truncated (84–95 %) with only a small part of the extracellular domain left. Hence, all are predicted to be loss of function alleles.

**Fig. 3:**
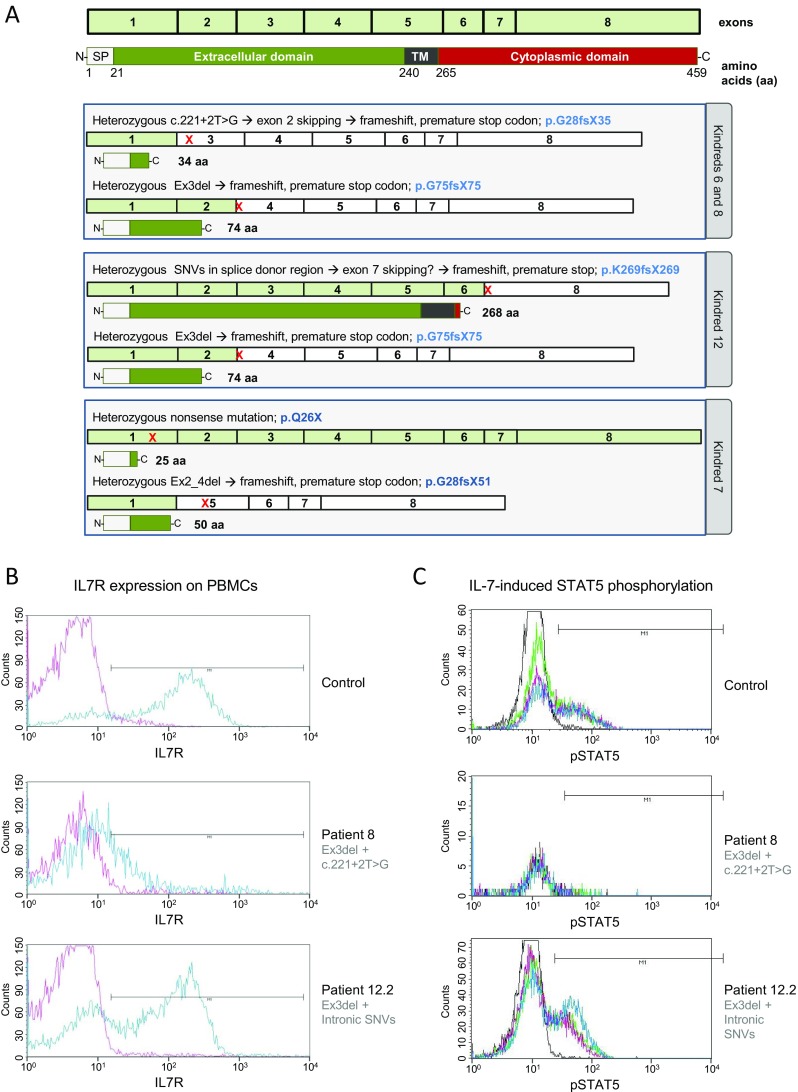
The impact of the mutations on IL7R expression and IL-7 signaling. **a** Schematic showing expected effect of the mutations on protein expression. If, as the phenotype suggests, the mutations are in a compound heterozygous setting, no patient would express full-length IL7R. **b** IL7R expression was measured by flow cytometry on PBMCs from a healthy control, patient 8 and patient 12.2. **c** STAT5 phosphorylation after stimulation with IL-7 (*red*), IL-2 (*green*) and IL-15 (*blue*) for 10 min was assessed by intracellular staining using whole blood or PBMCs from a healthy control, patient 8 and patient 12.2

In patients 6.2, 7.2 and 8, the heterozygous exonic deletion we identified by WES accompanied a heterozygous SNV in *IL7R* already identified by conventional diagnostic means and predicted to be deleterious. We could not confirm compound heterozygosity, as parental DNA was not available, but the apposite phenotypes of the patients imply that the predicted pathogenic *IL7R* mutations found here are biallelic. In the case of patient 8, cryopreserved PBMCs were available for functional testing that confirmed absence of IL7R expression and failure of STAT5 phosphorylation in response to IL-7 (Fig. 3c). This confirms the pathogenic nature of each allele, i.e. both the heterozygous exon 3 deletion and the heterozygous exon 2 splice donor site mutation c.221+2T>G produce loss of function (Fig. 3b).

Of the three SNVs in the exon 7 splice donor site of patient 12.2, c.876+6T>G is predicted to have the strongest effect, with Human Splicing Finder suggesting a broken donor site, and BDGP detecting the splice donor site with a score of 0.46, which is just above the threshold level of 0.4. The confidence of NetGene2 that a splice donor site is present drops from 0.81 for the wild-type sequence to 0.62 for the region with all three SNVs. However, the patient expressed IL7R at similar levels to a healthy control (67 % patient vs. 78 % control) (Fig. 3b) and sustained normal IL-7-induced STAT5 phosphorylation (39 % in both patient and control) (Fig. 3c). Thus, either the SNVs do not affect splicing or they are on the same allele as the exon 3 deletion. Our molecular findings for kindred 12 do not therefore explain the patients’ phenotype and emphasize the importance of confirmatory functional testing for variants of unknown significance.

Interestingly, in all patients that had a heterozygous *IL7R* mutation detected by prior analysis, we found a complementary heterozygous *IL7R* exon(s) deletion (Fig. 1), emphasizing the importance of looking for CNVs in *IL7R* in such cases. In total, about a quarter of our patients (5/19, 26 %) had such compound heterozygous *IL7R* deletions, with an additional patient (12.2) being a carrier. In our cohort, the exon 2 splice donor site mutation (c.221+2T>G) was most frequent, being present in six alleles, followed by p.Q26X and Ex3del, which were present in three alleles each.

## Discussion

In recent years, the discovery of PID-causing genes has accelerated markedly due to advanced sequencing technologies. Achieving a molecular diagnosis for individual patients with PID can, however, still prove difficult even if the disease is well defined and disease-causing genes are known. In our center, one such disease was T-B+NK+ SCID. New patients were directly analyzed by WES. Some patients were screened by a targeted PID NGS panel that had not yet incorporated the detection of CNVs. Older patients, for whom these technologies were not available at the time of presentation, were only analyzed by Sanger sequencing. Of these, five patients did not obtain a molecular diagnosis because only a single heterozygous nonsense or splice site mutation in *IL7R* was found. Only when we applied WES with CNV detection by ExomeDepth were we able to identify the cause of the disease in these five patients, through detection of a heterozygous single- or multi-exon deletion in *IL7R* as the second hit. Recently, Bayer et al. applied WES together with custom-designed chromosomal microarray to detect compound heterozygous mutations in *IL7R*, which also included a heterozygous deletion of exon 3 [24]. We found three kindreds (at least two alleles) with a deletion of exon 3, and one with a deletion of exons 2-4, suggesting a deletion hotspot involving exon 3.

At both deletion breakpoints that we mapped, we detected a microhomology of 2 and 3 bp, respectively (Figure S1 and Table S2). Short stretches of 2–30 bp of microhomology at the breakpoint sites are common in CNVs, with 70–80 % of human deletion CNVs showing microhomology [25, 26]. Interestingly, a third of microhomology stretches found in rare pathogenic microdeletions were 2 or 3 bp long [26]. Formation of microhomology-associated microdeletions is thought to be mediated by replication-based mechanisms such as non-homologous end-joining (NHEJ), microhomology-mediated end-joining (MMEJ) and fork stalling and template switching (FoSTeS) [26]. In addition to the microhomology, 83 % of deletion CNVs have at least one breakpoint within a known repetitive element [26]. The analysis of the four breakpoint regions in our patients showed this to be true for our deletion breakpoints too (Table S2). Breakpoint region 1 of the exon 3 deletion is within a long interspersed nuclear element/L1 repeat, whereas breakpoint region 2 of the exon 2-4 deletion is within a small interspersed nuclear element/Alu repeat. The local genomic architecture in parts of the *IL7R* gene may thus render it particularly susceptible to deletion CNVs.

We found that single- or multi-exon deletions in *IL7R* are relatively frequent disease alleles in autosomal recessive T-B+NK+ SCID and can be detected by CNV analysis. Without the application of methods specifically to scrutinize CNVs, these disease alleles would be missed in the heterozygous state. Our cohort suggests that CNV analysis would be required to reach a molecular diagnosis in about a quarter of patients. Especially for centers that already use WES or targeted gene panels, we suggest they extend their analysis to include CNV detection. Various techniques are already established so can be applied without much difficulty. Coverage of *IL7R* by available whole exome platforms is sufficient to call such variants using either ExomeDepth or one of the other CNV calling methods summarized in Tan et al. [2]. The same applies for targeted gene panels that include *IL7R*; at Great Ormond Street Hospital, we now use ExomeDepth for CNV detection in samples analyzed with the targeted primary immunodeficiency gene panel. Alternatively, MLPA analysis could be employed, as is routinely done in screening for Artemis deficiency [27].

## Electronic supplementary material

Below is the link to the electronic supplementary material.

ESM 1 (DOCX 1867 kb)
