# Supplementary material for: Identification of Heterozygous Single- and Multi-exon Deletions in IL7R by Whole Exome Sequencing
Source: J Clin Immunol. 2016 Nov 2;37(1):42–50. doi: 10.1007/s10875-016-0343-9 (PMC5226981; doi:10.1007/s10875-016-0343-9)
Supplement: Supplementary file 1 — (DOCX 1867 kb) [file 10875_2016_343_MOESM1_ESM.docx]

**SUPPLEMENTAL DATA**

**Identification of heterozygous single- and multi-exon deletions in *IL7R* by whole exome sequencing**

**Methods**

**PCR and sequencing analysis.** Specific primers were designed in Primer3web version 4.0.0 (http://bioinfo.ut.ee/primer3/). Primer sequences are available on request. Capillary sequencing was performed according to standard methods. Sequences were aligned with the consensus coding sequence (human genome assembly 38) in nucleotide BLAST (http://blast.ncbi.nlm.nih.gov/blast/). ChromasLite Version 2.1.1 was used for visualization of the sequences.

**Breakpoint characterization.** The sequences of the introns harboring the breakpoints were analysed for repetitive elements using the online database RepeatMasker (<http://www.repeatmasker.org/>).

**Splice site mutation predictions.** The intronic mutations were analysed for their impact on splicing using the online prediction programmes Human splicing finder (<http://www.umd.be/HSF3/>), NetGene2 splice site prediction (<http://www.cbs.dtu.dk/services/NetGene2/>) and BDGP splice site prediction (<http://www.fruitfly.org/seq_tools/splice.html>).

**Figures**

**Figure S1**

Patient 7.2

Intron 1-2

Intron 4-5


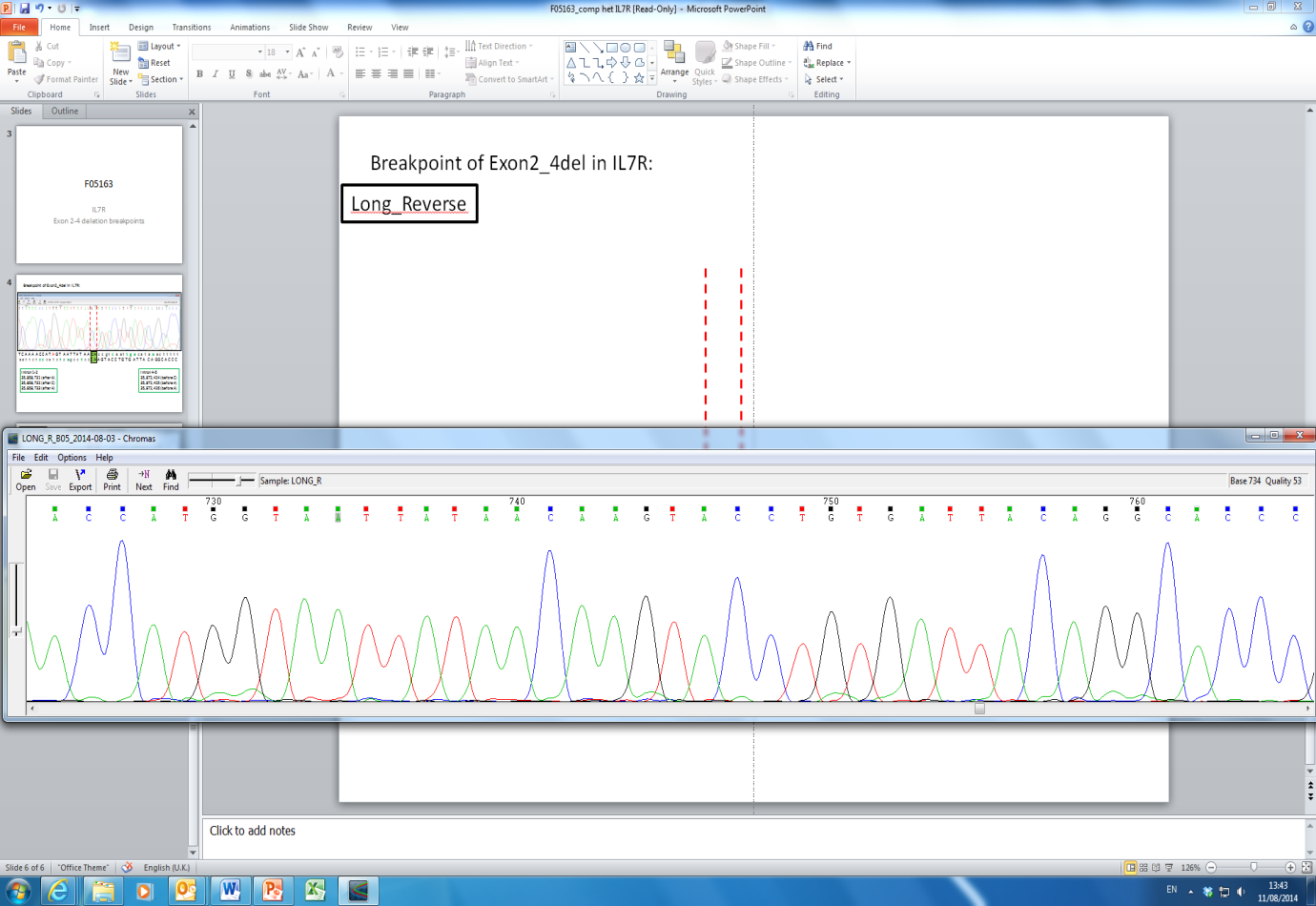


ACCATGGTAATTATAACAAGTACCTGTGATTACA

*TCCCATCTCAGCCTCC*CAAGTACCTGTGATTACA

13,693 bp deletion

1-2 or

4-5

ACCATAGTAATTATAACA*CCGTCAATTGACATAA*

Intron 1-2

Intron 4-5

35,872,325

35,858,629

Patient 6.2

Intron 2-3

Intron 3-4


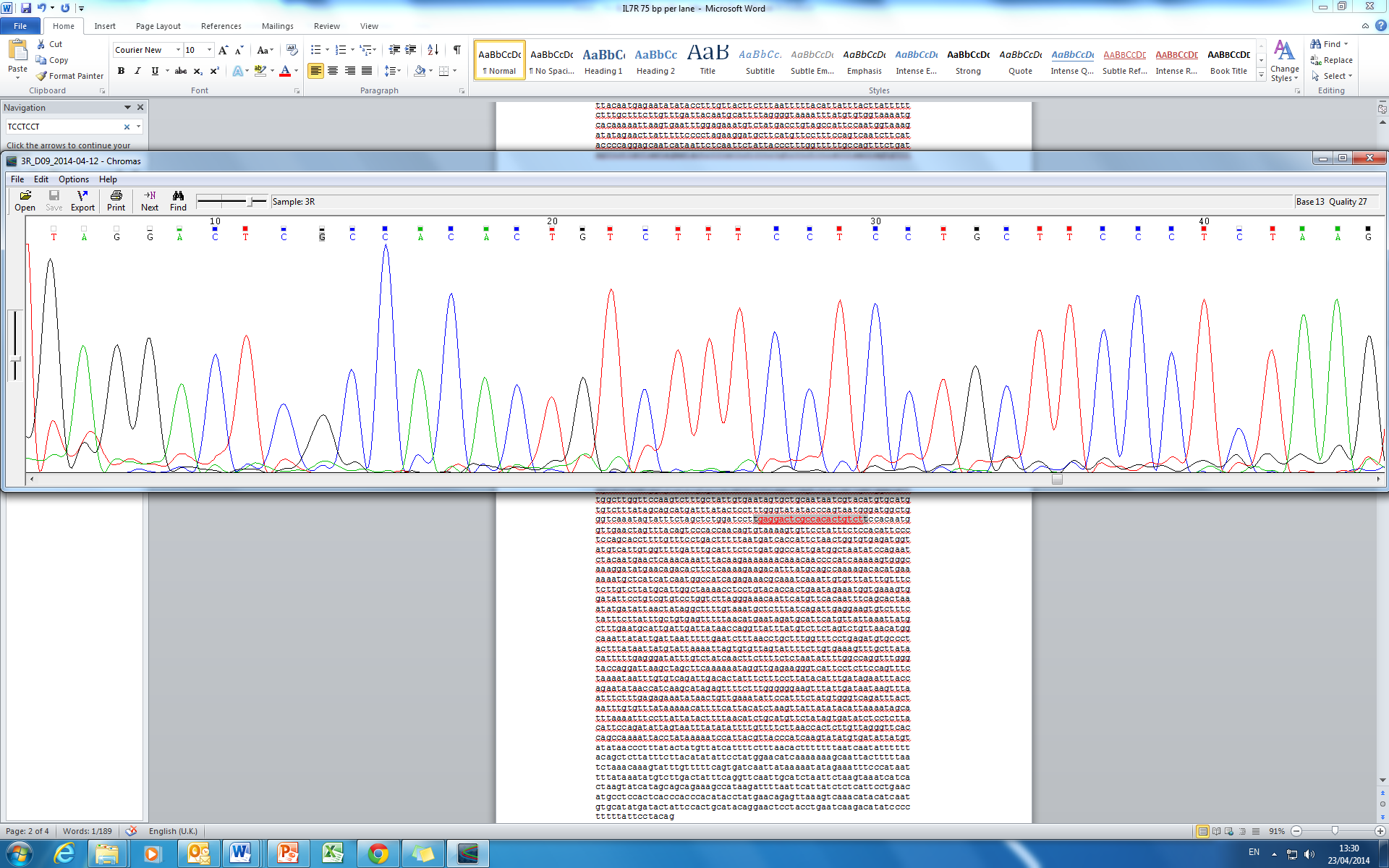


4,253 bp deletion

GGACTCGCCACACTGTCTTTCCTCCTGCTTCCCT

GGACTCGCCACACTGTCTT*CCACAATGGTTGAAC*

*GTTCTCCACCCACTGC*CTTTCCTCCTGCTTCCCT

Intron 2-3 or

intron 3-4

Intron 2-3

Intron 3-4

35,869,796

35,865,539

**Figure S1: Deletion breakpoints.** Shown are Sanger sequencing results of patients 6.2 and 7.2. Indicated are the patient sequence compared to reference intronic sequences; the 3 or 2 bases of microhomology (in red) that could belong to either intron 2-3 or 3-4, or intron 1-2 or 4-5, respectively; the size of the deletion; and the chromosomal location of the last and first definite base in the respective intron. Deletion breakpoints in families 8 and 12 could not be determined, suggesting that they are different to family 6.

**Table S1: Predictions about the impact of the splice site variants.**

|  | Human Splicing Finder | BDGP splice site prediction | NetGene2 splice site prediction |
| --- | --- | --- | --- |
| Exon 2 splice donor site | | | |
| wt | - | Score: 0.97 | Confidence: 0.94 |
| c.221+2T>G | Broken WT donor site; most probably affecting splicing. | None found* | None found* |
| Exon 7 splice donor site | | | |
| wt | - | Score: 0.97 | Confidence: 0.81 |
| c.876+6T>G  c.876+12T>G  c.876+15T>G | Broken WT donor site; most probably affecting splicing.  Activation of intronic cryptic donor site; potential alteration of splicing. | Score: 0.46 | Confidence: 0.62 |

*Detection threshold: 0.4

c.876+6T>G alone: BDGP – 0.46; NetGene2 – 0.65

c.876+12T>G alone: BDGP – 0.46; NetGene2 – 0.82

c.876+15T>G alone: BDGP – 0.46; NetGene2 – 0.70

**Table S2: Characteristics of the deletion breakpoints.**

|  |  | Exon 3 deletion | Exon 2-4 deletion |
| --- | --- | --- | --- |
|  | Start (GRCh38.p3) | Chr5: 35,865,543 | Chr5: 35,858,632 |
|  | End (GRCh38.p3) | Chr5: 35,869,795 | Chr5: 35,872,324 |
|  | Size (bp) | 4,253 | 13,693 |
|  | Microhomology (bp) | 3 | 2 |
|  | Sequence* | …TGTCTTccaca…tgccttTCCTC… | …ATAACAccgtc…tcccaAGTAC… |
| Breakpoint region 1 | repetitive element | L1PA5 | - |
|  | repeat class/family | LINE/L1 | - |
|  | length of repetitive element (bp)  (before and after breakpoint) | 755  (582, 173) | - |
| Breakpoint region 2 | repetitive element | - | AluSx1 |
|  | repeat class/family | - | SINE/Alu |
|  | length of repetitive element (bp)  (before and after breakpoint) | - | 283  (115, 168) |

*The sequence remaining in the patients is represented by capital letters, the deleted sequence by small letters. Nucleotides of the microhomology are underlined.

Abbreviations: LINE, long interspersed nuclear element; SINE, short interspersed nuclear element.
